# Editorial: Biointerfacing 2D Nanomaterials and Engineered Heterostructures

**DOI:** 10.3389/fbioe.2020.639723

**Published:** 2021-01-15

**Authors:** Alfredo Maria Gravagnuolo, Eden Morales-Narváez, Alessandro Martucci

**Affiliations:** ^1^Division of Pharmacy and Optometry, School of Health Sciences, Faculty of Biology Medicine and Health, & National Graphene Institute, The University of Manchester, Manchester, United Kingdom; ^2^Biophotonic Nanosensors Laboratory, Centro de Investigaciones en Óptica A. C., León, Mexico; ^3^Dipartimento di Ingegneria Industriale, Università di Padova, Padova, Italy

**Keywords:** nanomaterials, biointerfaces, biological interactions, nanotoxicology, nanodevices, nanobiotechnology, tissue engineering

A new rising area of research known as nano-bio-technology has emerged in recent years. This research addresses the challenges faced by society via wide inter-disciplinary approaches, encompassing a wide variety of scientific sub-domains such as nanoscience, chemistry, and biology. Progress in these fields requires awareness of nano-bio-technology and an understanding of how nanomaterials interact with simple biomolecule models and complex biological systems. This Research Topic has been in development since 2017 and forms part of a longer-term process of knowledge gathering that aims to bring together disparate experiences and expertise in smart nano-bio-enabled technologies. *Frontiers*, with its cross-sectoral approach to publication, provides an ideal platform for both undertaking and presenting this inter-disciplinary project. This Research Topic, the first of a series on the subject, focuses on nano-biotechnology based on 2D nanomaterials and their engineered heterostructures.

Graphene and other two-dimensional (2D) nanomaterials hold great promise for the future of biotechnology and biomedicine. In this context, the biological interfacing of 2D materials is a key step to improve their biocompatibility, biofunctionality, and selectivity toward biotechnological applications such as biosensors, microchip bioreactors, biological fuel cells, biocatalysis, bioremediation, nanomedicine, and bioelectronics.

Exploring the extent to which 2D nanomaterials can be integrated into biotechnology, the articles collected in this Research Topic aim to elucidate cellular and molecular responses to 2D nanomaterials. Significant new knowledge has been accumulated since 2004 when graphene was first isolated by the Manchester-based Russian scientists Sir Andre Geim and Sir Konstantin Novoselov, who won The Nobel Prize for Physics for this breakthrough in 2010.

On the one hand, this topic aims to summarize and describe the biological interactions of carbon-based nanomaterials through reviews such as those of Rahmati and Mozafari and Palmieri, Sciandra et al.. On the other hand, we also aimed to explore the wider field, with new insights about nano-bio-interfaced graphene, graphene oxide, and MoS_2_ presented in original research by Palmieri, Di Pietro et al., Bernabò et al., Trapani et al., and Singh et al..

Rahmati and Mozafari also review cellular and molecular responses to carbon-family nanomaterials from 0D fullerenes and carbon quantum dots (CDs), to 1D carbon nanotubes (CNTs), 2D graphene, 3D diamond-like carbon (DLC), and mesoporous carbon materials. They highlight the physicochemical properties of graphene to affect immunological cellular and molecular responses at the nano-bio-interface. The success of *in vivo* applications (i.e., implanted biomedical devices and drug delivery systems) depends on immunological interaction with the human body.

“*It is expected that the most recent perspective strategies for improving the biological responses to carbon-based nanomaterials can revolutionize their functions in emerging biological applications”* (Rahmati and Mozafari).

Due to the excellent mechanical, electrical, and surface properties of graphene-based materials, they are used to interact with a wide portfolio of synthetic and natural polymers, mimicking native musculoskeletal tissue to fabricate so-called 3D graphene scaffolds, as explored in the mini-bibliographic review conducted by Palmieri, Sciandra et al.. Functionalized graphene-related materials and composites can stimulate stem cell differentiation and muscle contraction due to their electrical conductivity. 2D materials have been studied for cardiac, neuronal, bone, skin, adipose, and cartilage tissue regeneration. The review covers both the use of natural and synthetic polymers and some of the different techniques available for the fabrication of such 3D scaffolds. It concludes with a discussion of the biological performance (mainly *in vitro*) of these devices for muscle regeneration.

The *original research articles* included in this Research Topic, by Palmieri, Di Pietro et al., Bernabò et al., and Trapani et al., shed light on graphene oxide biointerfacing at molecular levels for biomedical applications, with particular attention to nucleic acids, dipeptides, lipids (and lipid bilayers), which are also affected by divalent cations. These important findings will inspire future use of “smart” graphene oxide (GO) nanosheets in nanomedicine, for drug delivery and diagnostics.

In the study by Palmieri, Di Pietro et al., GO with trapped divalent cations such as calcium and magnesium, are proposed as a nano-concentrator for facilitating the enhanced sensitivity of sequencing platforms by short RNA enrichment, thereby, offering innovative methods to improve liquid biopsy performance and contributing to future approaches to RNA detection. This innovative study changed pH to tune the desorption of nucleic acids in a consistent fashion.

“*After nano-concentration, small RNAs can be selectively released from surface by desorption induced at high pH in the presence of Mg*^2+^ *ions. Conversely, with calcium, all RNA species are released*” (Palmieri, Di Pietro et al.).

Importantly, when compared with the gold standard in promoting *in vitro* fertility of mice spermatozoa, GO was also revealed to improve *in vitro* fertilization (IVF) outcomes, thus offering new opportunities and capabilities in the treatment of human infertility (Bernabò et al.). In their contribution, Bernabò et al. concluded that upon exposure of spermatozoa to the GO, the nanomaterial works as a cholesterol extractor to modify membrane fluidity, preserving sperm function via improved membrane-protein interactions, which in turn improved IVF.

Trapani et al. provided further insights on GO-lipid bilayer (as cell membrane model) interactions for nano-biotechnological applications. Decoration of GO nano-sheets, with aromatic dipeptides, with and without divalent copper cations, was investigated as a nano-tool to affect the viscoelastic properties and fluidity of the membrane. This method can tune the interactions of the nanomaterial with lipid bilayer models and eventually with cancer cells, as demonstrated by proof of principle experiments on two cancer cell lines, for potential theragnostic platforms.

Singh et al. describe a methodology for the production and characterization of 2D nanomaterials, and the modality of exposure of pathogens for use as a disinfectant. The interaction of 2D nanosheets made of MoS_2_ and GO with bacteria and viruses highlighted that the antibacterial and antiviral action of GO and MoS_2_ nanosheets may depend on the surface properties of the two nanomaterials and biological targets (Singh et al.). The impact of MoS_2_ (stronger antibacterial) and GO (stronger antiviral) was reversed.

“*The experimental results in viruses are really novel and somewhat surprising, evidencing a stronger antiviral action of graphene oxide as compared to MoS*_2_” (Singh et al.).

Exploring this new research field is an exciting journey in unknown multidimensional terrain, not unlike “the flatland” imagined in the novel by Edwin A. Abbott, as remarked by A. Gaim and K. Novosolov in their articles and lectures. We anticipate the significant impact of novel 2D nanomaterials on nano-biotechnology over the next 10–15 years. Pioneering studies on new 2D materials beyond graphene engineered van der Waals heterostructures and bio-hybrid nanoassemblies will create new opportunities. However, for real-world application, the cytotoxicity, large scale production, and the high-throughput quality assessment of nano-objects should be investigated. Upcoming smart nano-bio-enabled devices with unprecedented tunable properties that are not available in nature could be used to address the numerous problems faced by modern society. From this perspective, we hope this Research Topic contributes valuable insights into this exciting field.

## Author Contributions

AG and EM-N conceived the idea and coordinated the Research Topic. AG, EM-N, and AM co-edited this Research Topic and approved the submitted version of the Editorial Article. All authors contributed to the article and approved the submitted version.

## Conflict of Interest

The authors declare that the research was conducted in the absence of any commercial or financial relationships that could be construed as a potential conflict of interest.

